# Intranasal endoscopic prelacrimal recess approach for maxillary sinus inverted papilloma

**DOI:** 10.1007/s00405-018-5078-1

**Published:** 2018-08-04

**Authors:** Qian-Qian Yu, Ge Guan, Nian-Kai Zhang, Xiao-Wen Zhang, Yan Jiang, Yuan-Yuan Lian, Ting-Ting Liu, Xiao-Dan Jiang, Na Li

**Affiliations:** 1 Department of Otolaryngology, Affiliated Hospital of Qingdao University, Qingdao, China; 2 Center of Organ Transplantation, Affiliated Hospital of Qingdao University, Qingdao, China

**Keywords:** Transnasal endoscopic prelacrimal recess approach, Maxillary sinus, Inverted papilloma, Nasal cavity, Paranasal sinuses

## Abstract

**Purpose:**

This study aims to determine the indications and effectiveness of transnasal endoscopic prelacrimal recess approach (PLRA) in patients with maxillary sinus inverted papilloma (IP).

**Methods:**

We retrospectively analyzed 71 patients treated in our institution for maxillary sinus IP between August 2008 and April 2015. 20 patients underwent endoscopic surgery via PLRA. All the patients who had postoperative follow-up for 3 years were enrolled. Demographic data, surgical technique, location of IP attachment, intra- and postoperative complications, follow-up duration and recurrence were recorded.

**Results:**

The outpatient follow-up period was 3–10 years after surgery. Recurrence of IP was seen in 6 (8.5%) of 71 patients, including 1 patient in the PLRA group. The recurrence rate was 5% in the PLRA group. Six of 71 patients experienced postoperative complications, but none was observed in the PLRA group.

**Conclusion:**

Transnasal endoscopic PLRA is a minimally invasive, safe and effective method for maxillary sinus IP. The indication for PLRA is tumor pedicle located on the antero-inferior or infero-lateral wall or at multiple attachment sites of the maxillary sinus.

## Introduction

Inverted papilloma (IP) of the nasal cavity and paranasal sinuses is a benign tumor that accounts for 0.5–4% of all sinonasal tumors [1]. Two features of sinonasal IP are especially noteworthy: (1) it has a high propensity toward recurrence, with a recurrence rate of 5–30%; and (2) it is associated with squamous cell carcinoma in 5–21% of patients [2]. Therefore, aggressive surgical excision is the recommended treatment option. The maxillary sinus is the most frequent site of tumor origin (26–46.4%) [3–5]. Despite advances in surgical techniques, the surgical approaches utilized to address tumors occurring in the maxillary sinus remain controversial. Because of the facial incision, the extranasal approach has a long healing time and inevitably forms scars after surgery, which has a negative impact on quality of life. With the development of nasal endoscopic and high-resolution imaging techniques, nasal endoscopic surgery has become the most commonly used treatment for these diseases [6, 7]. However, for anatomical reasons, the positions of the anterior and medial walls and the alveolar crypt of the maxillary sinus are not easily visible and manageable, therefore, it is difficult to perform the resection using the traditional endoscopic approach. Endoscopic medial maxillectomy is currently the gold standard for treatment of maxillary sinus IP. However, the procedure has numerous complications, such as epiphora, incrustation, and inability to feel the nasal airflow, due to resection of the nasolacrimal duct (NLD) and the inferior turbinate (IT). In 2007, Zhou et al. [8, 9] proposed the intranasal endoscopic prelacrimal recess approach (PLRA) to the maxillary sinus. This provides wide access to the walls and recesses of the maxillary sinus, while the IT and NLD are preserved [10]. 6 years ago, we adopted the PLRA to address maxillary sinus IP to avoid an external incisional wound. We performed a retrospective analysis of patients treated in our institution for maxillary sinus IP between August 2008 and April 2015. We aimed to determine the indication and effectiveness of transnasal endoscopic PLRA in patients with maxillary sinus IP.

## Methods

### Patients

**Fig. 1:**
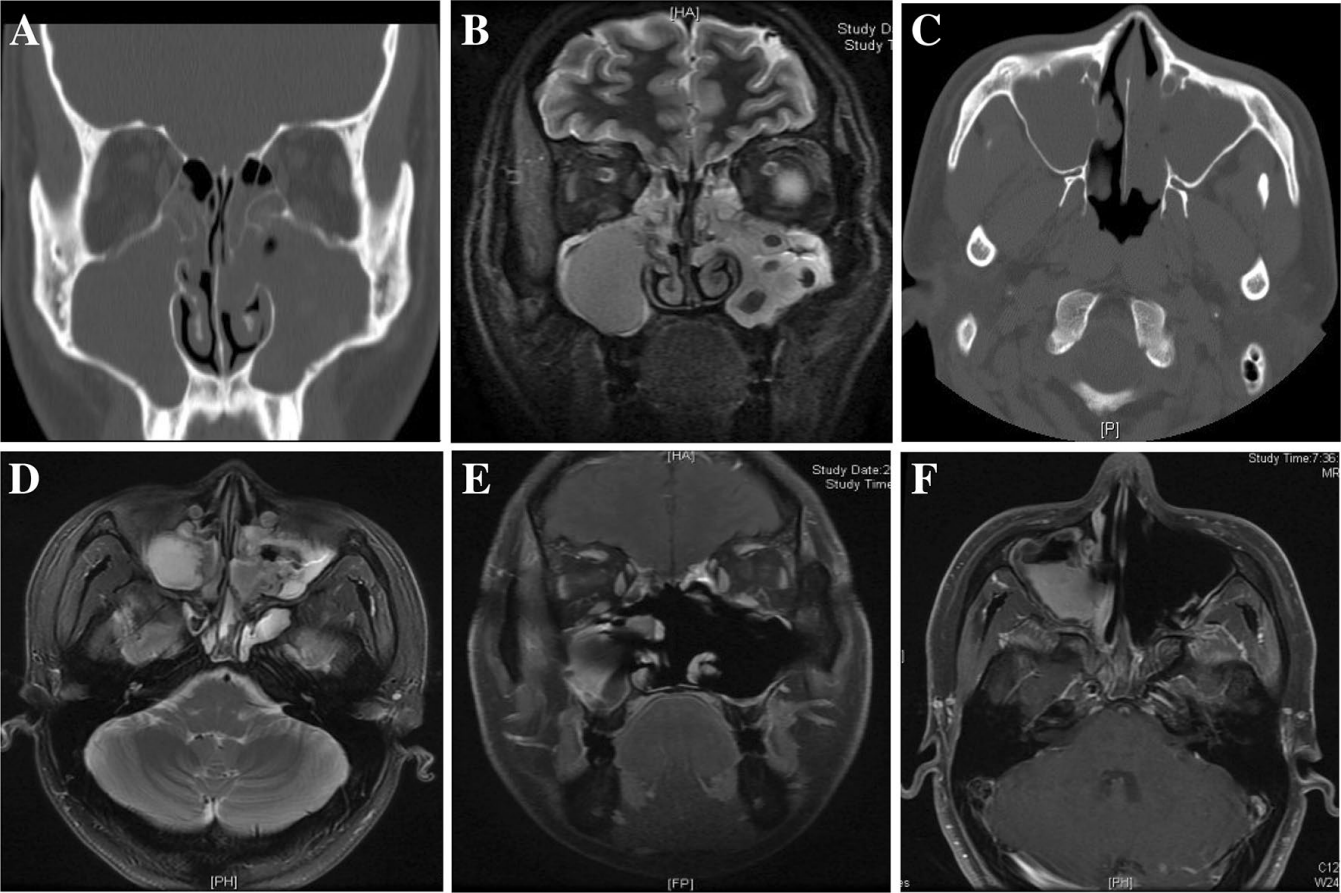
Preoperative and postoperative paranasal sinus CT and MRI of IP of maxillary sinus. **a** Coronal CT demonstrated opacification of the bilateral maxillary sinus. **b** Coronal T2-weighted MR image with contrast showed IP as an intermediate and irregular mixed signal intensity mass of the left-side maxillary sinus. **c** Axial CT demonstrated opacification of the bilateral maxillary sinus. **d** Axial T2-weighted MR image showed the tumor lying around the left-side maxillary sinus. **e** Postoperative coronal MRI showed a clear left and right maxillary sinus with thickened mucosa and no tumor recurrence. **f** Postoperative axial MRI showed a clear left and right maxillary sinus with thickened mucosa and no tumor recurrence

This retrospective study was performed in 71 patients with histopathologically confirmed maxillary sinus IP who underwent surgery at the Department of Otolaryngology, Affiliated Hospital of Qingdao University, Shandong Province, China from August 2008 to April 2015. This study was approved by the Institutional Review Board of our hospital. Only adult patients with maxillary IP and with at least 3 years follow-up were considered eligible for inclusion. Patients with other sinonasal localizations of IP were excluded, along with patients with concomitant squamous cell carcinoma. All patients had preoperative computed tomography (CT) and magnetic resonance imaging (MRI) scans (Fig. 1), histopathological examination and outpatient assessment with nasal endoscopy. Demographic data, surgical technique, location of IP attachment sites, intra- and postoperative complications, follow-up duration, and recurrence were recorded.

### Surgical technique

In all cases, surgery was performed under general anesthesia. Nasal mucosal blood vessels were contracted with 0.01% epinephrine gauze. Endoscopic examination confirmed the extent of the lesion. The endoscopic resection of the uncinate process, open and enlarge the ostium of the maxillary sinus. If pathological results were not obtained before surgery, the neoplasm in the maxillary sinus was taken for pathological examination by frozen section during surgery. After the pathological diagnosis of IP was established, the surgical approach was determined based on CT findings and the extent of the lesion seen during surgery. PLRA was selected for complicated IPs involving the alveolar crypts, prelacrimal recess, and antero-medial-inferior walls of the maxillary sinus, or with two or more root pedicles and with multiple areas of bone destruction in each wall of the maxillary sinus.

**Fig. 2:**
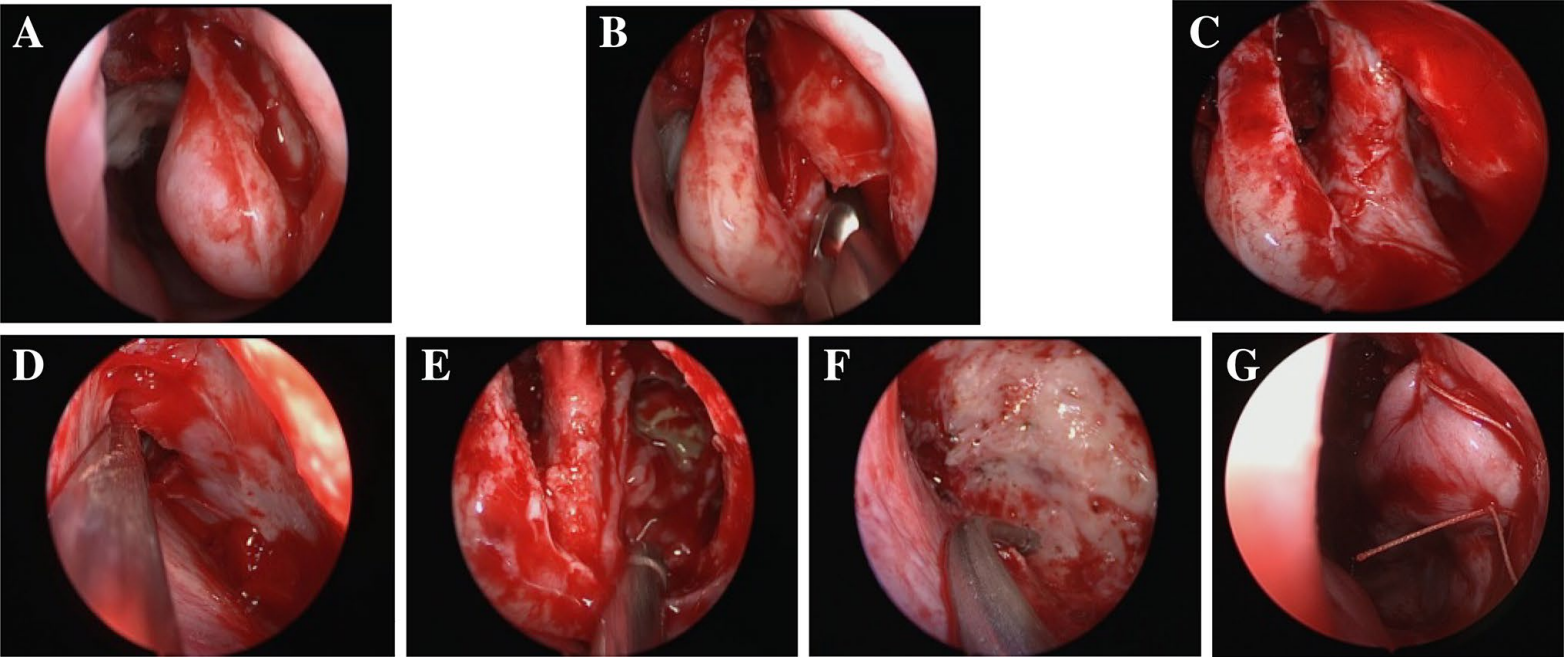
Surgical procedures for the PLRA. **a** Curved mucosal incision on the lateral wall of the nasal cavity. **b** Mucoperiosteal flap was elevated to show lacrimal bone and inferior turbinate. **c** Nasolacrimal duct was released from its canal. **d** Antero-medial wall of maxillary sinus was exposed and the recess was widened. **e** Maxillary sinus was entered through the antrostomy made at the prelacrimal recess. **f** The tumor was removed completely, and the tumor base was drilled. **g** IT-NLD mucosal flap was repositioned and the incision was sutured

PLRA included the following surgical steps. The incision was infiltrated with 1% lidocaine with 1:100,000 epinephrine solution. A curved mucosal incision on the lateral wall of the nasal cavity was made between the anterior aspect of the inferior turbinate and the edge of the pyriform aperture to the bone (Fig. 2a). The mucosa from the subperiosteal level was elevated posteriorly to the insertion site of the inferior turbinate concha and then the bony attachment of IT was disconnected. The bony inferior orifice of NLD could be seen after the mucoperiostium was elevated posteriorly (Fig. 2b). We chiseled off the anterior bony portion of the medial wall of the maxillary sinus (part of the maxillary frontal process), and after chiseling the bone posteriorly, the NLD was exposed and the IT-NLD flap was formed (Fig. 2c). The IT-NLD flap was pushed medially and the antero-medial wall of the maxillary sinus was exposed (Fig. 2d). The maxillary sinus was entered through the antrostomy made at the prelacrimal recess (Fig. 2e). The maxillary sinus was exposed widely when the antrostomy was adequately enlarged, and all pathological tissues were removed under direct visualization (Fig. 2f). The IT-NLD mucosal flap was repositioned and the incision was sutured at the end of the operation (Fig. 2g).

### Follow-up

Patients were endoscopically evaluated at 2 week and 1, 2 and 3 months postoperatively, which included cleaning the crust and cysts, and then every 2–6 months, according to endoscopic findings and tailored to individual patient needs. Nasal irrigation with 0.9% sodium saline were also prescribed for at least 6 months. At the time of postoperative visit, if suspicious tumor tissue was found, histopathological biopsy or imaging examinations were performed timely.

## Results

From August 2008 to April 2015, 71 patients were treated in our department for maxillary sinus IP. The average age of the patients at initial diagnosis was 52 years (range 25–76 years), with a male-to-female ratio of 1.63:1 (44 men and 27 women). Symptom onset occurred 1 week to 15 years before admittance to the hospital. The most frequent presenting symptom was nasal obstruction. This was followed by rhinorrhea or with blood and a feeling of pressure. A total of 29 (40.8%) patients were considered to have had revised operation. The right side was more commonly involved than the left (38/33, 53.5%). Postoperative pathology was IP with mild dysplasia in 7 cases, moderate dysplasia in 2, and severe dysplasia in 1. According to the Krouse classification method [11], 28 cases were grade II and 43 were grade III. 14 patients underwent endoscopic surgery via middle meatal antrostomy (MMA) of the maxillary sinuses. 30 patients underwent surgery via endoscopic medial maxillectomy (EMM), which comprised 23 simple EMMs (medial maxillary wall was resected into its posterior two-thirds) and 7 extended EMMs (NLD and IT were removed); 7 patients underwent surgery via endoscopic MMA combined with Caldwell-Luc surgery; and 20 patients underwent endoscopic surgery via PLRA (Table 1). The patients underwent surgery via PLRA mostly because tumors were present in the antero-inferior or infero-lateral wall or multiple attachment sites of the maxillary sinuses (Table 2).

**Table 1:** Postoperative complications and recurrence rate

	MMA	MMA + LU	EMM (simple)	EMM (extended)	PLRA
No. of patients	14	7	23	7	20
Complications					
Epiphora	–	–	–	1	–
Periorbital swelling	–	–	1	–	–
Facial numbness	–	1	–	–	–
Epistaxis	1	–	1	–	–
Dry nose	–	–	–	1	–
Recurrence	2	1	2	–	1

**Table 2:** Patient demographic and clinical characteristics in the PLRA group

Patient no.	Sex	Age (years)	Origin, wall (side)	Revised operation	Follow-up (months)	Complications	Recurrence
1	M	46	MM, IM (R)	No	37	No	No
2	M	63	MM, IM (L)	No	37	No	No
3	M	52	AM (R)	No	39	No	No
4	M	58	MM (L)	No	41	No	No
5	F	62	AM, MM (L)	No	44	No	No
6	F	47	AM, MM (R)	Yes	44	No	No
7	F	76	MM, LM, IM (L)	Yes	46	No	No
8	F	51	MM, PM (L)	Yes	47	No	No
9	M	36	AM, MM (R)	No	49	No	8 months after surgery
10	M	51	MM, AM, IM (R)	No	51	No	No
11	M	78	MM, AM (R)	Yes	54	No	No
12	M	60	Whole wall (R)	No	55	No	No
13	M	37	LM, IM (R)	Yes	57	No	No
14	M	67	PM, IM (R)	No	57	No	No
15	M	73	AM, IM, MM (L)	Yes	58	No	No
16	F	57	Whole wall (L)	Yes	61	No	No
17	F	47	AM, MM, IM (R)	Yes	65	No	No
18	M	50	MM, IM (R)	Yes	68	No	No
19	M	56	MM, AM (L)	No	70	No	No
20	M	62	AM (L)	Yes	73	No	No

*SM* superior wall of the maxillary sinus, *PM* posterior wall of the maxillary sinus, *MM* medial wall of the maxillary sinus, *LM* lateral wall of the maxillary sinus, *AM* anterior wall of the maxillary sinus, *IM* inferior wall of the maxillary sinus, *R* right, *L* left

Postoperative follow-up period was 3–10 years, with an average of 5.5 years. Of the 71 patients, 6 (8.5%) had postoperative recurrence (Table 1). Four cases had recurrence within 2 years after surgery, and 1 within 5 years. Among the 20 patients with PLRA, 1 had recurrence (Table 2), in which the lesion was located on the anterior-medial wall of maxillary sinus cavity and recurred 8 months after surgery. During reoperation by nasal endoscopy, expanded resection of the lesion was performed and the bone at the base of the margin was adequately treated, and there was no recurrence after follow-up for 41 months.

6 of the 71 patients had postoperative complications (Table 1). After surgery by PLRA, there was no vision disorder, diplopia or epiphora; no facial numbness, pain or swelling; and no nasal complications such as dry nose. During surgery, the tumor was found to involve the NLD in one case. Thus, while preserving a 5-mm margin, the tumor tissue and the middle and lower parts of the NLD were resected together, and dacryocystorhinostomy was performed. At re-examination at 3 months after surgery, the operated cavity was completely epithelialized and no tumor recurred. In 4 cases, cystic vesicles or granuloma hyperplasia occurred in the operated cavity, which were removed by nasal endoscopy, and disappeared 2–4 weeks after flushing the nasal cavity, and no recurrence was found. Stenosis of the middle meatal antrostomy and scarification were observed in 2 IP cases.

## Discussion

Due to the restriction of the maxillary sinus anatomy and the characteristics of tumor biological behavior, the recurrence rate of maxillary sinus IP after surgery under nasal endoscopy is still high. IP that originates in the maxillary sinus, due to the delayed appearance of symptoms, has a relatively great extent of tumor. Our experience shows that it is important to search carefully for and locate the origin of the tumor under nasal endoscopy. The tumor origin of IP in the nasal cavity and sinuses has a high degree of consistency with bone hyperplasia in CT imaging [12], and the precise location of the lesion can be determined in conjunction with endoscopic findings. Choosing a reasonable surgical procedure based on the location and extent of the lesion reduces both the recurrence rate and surgical complications.

In the present study, 14 patients with the bases of tumors confined to the posterior-lateral wall of the maxillary sinus, but far posteriorly, underwent surgery with expanded MMA. Two cases had recurrence after surgery, but no obvious complications were observed. For the patients with IP that originated in the medial wall of the maxillary sinus with significant bone destruction, poor differentiation, or dysplasia, complete resection of the medial wall of the maxillary sinus was performed. This approach can expose the entire maxillary sinus and completely remove the tumor, but the need to remove the IT and NLD results in major trauma. In the present series of patients, one had epiphora and another had dry nose. Seven patients whose tumor was located in the anterior, medial and inferior walls of the maxillary sinus, underwent MMA combined with Caldwell-Luc surgery. After fully expanding the natural opening of the maxillary sinus, an incision in labiogingival groove was made, but postoperative complications such as facial numbness or tooth pain were recorded. According to our experience, the lesions are fully exposed by this approach, but the wounds are larger. This approach has gradually been replaced by the PLRA in our department.

Studies have been carried out in the surgical approach for this disease. Weber et al. [13] reported a case series of 12 patients with IP of the maxillary sinus (Krouse II–III) who were endoscopically treated with medial maxillectomy with IT preservation. There was no recurrence of tumor after 12–80 months. Nakamura et al. [14] reported a method with the preservation of the NLD during EMM for IP. However, if the tumor was attached to the bottom of maxillary sinus with irregular prominences, it was difficult to manage intranasally by this method. Tewfik et al. [15] recommended canine fossa puncture to remove the complex lesions in the maxillary sinus cavity. Because the ring drilling point is located on the outer tip of the root of the canine tooth and the upper alveolar nerve may be damaged, there may still be complications after surgery, such as numbness in the upper lip and cheeks, numbness of the teeth, and cheek pain and swelling.

The advantages of PLRA are that it can be performed completely in the nasal cavity. Nasal endoscopy at 0° can obtain a good visual field; the lesions in the maxillary sinus are fully exposed, leaving no blind angle, which facilitates complete removal of the lesions. Through displacement, the NLD and IT are protected and the function of the nasal cavity and NLD is thus maintained. At the same time, PLRA can avoid additional incisions, and avoid damage to the anterior maxilla wall and to the infraorbital nerve and upper alveolar nerve. This also avoids postoperative occurrence of other complications such as pain and swelling in the upper lip and cheek, tooth numbness, and cheek numbness. However, the disadvantage is that the NLD needs to be protected during surgery. Therefore, there is a high need of surgeon’s anatomical knowledge and proficiency in nasal endoscopic surgery.

Conclusion, the key to successful surgery by the PLRA lies in the following aspects. The extent of the lesion needs to be confirmed and the operative field needs to be managed. Imaging should be performed before surgery to determine the extent and features of the lesion. We are in favor of performing MMA before PLRA because it provides a better drainage route for the maxillary sinus and wider access to the maxillary sinus for postoperative treatment. During surgery, the tumor tissue and surrounding mucosa should be removed together for the better prevention of postoperative recurrence. The bone hyperplasia in the basal part of the tumor needs to be removed with a bone chisel or drill [16]. The occurrence of adverse reactions must be avoided during surgery [17]. The PLRA achieves the exposure range of medial maxillectomy and the Caldwell-Luc surgery, and retains the integrity of the IT and lacrimal duct system. While fully exposing the tumors for complete removal, the nasal function is well protected. In most cases, the PLRA can replace the Caldwell-Luc surgery and is an ideal surgical method in treating maxillary sinus IP under endoscopy. The sample size in our study was small, and a larger group with extended follow-up is required to verify the effectiveness of the protocol.
